# Magnitude and its associated factors of gestational trophoblastic disease in East African countries, systematic review and meta-analysis

**DOI:** 10.3389/fonc.2025.1515246

**Published:** 2025-05-29

**Authors:** Nigusie Abebaw, Gebeyaw Biset, Nega Yimer, Hailemariyam Gezie, Moges Workneh, Chalie Mulugeta, Tadele Emagneneh, Abebaw Alamrew, Zelalem Birhan

**Affiliations:** ^1^ Department of Midwifery, College of Medicine and Health Science, Wollo University, Dessie, Ethiopia; ^2^ Department of Pediatrics and Child Health, College of Medicine and Health Science, Wollo University, Dessie, Ethiopia; ^3^ Department of Comprehensive Nursing, College of Medicine and Health Science, Wollo University, Dessie, Ethiopia; ^4^ Department of Emergency and Critical Care Nursing, College of Medicine and Health Science, Wollo University, Dessie, Ethiopia; ^5^ Department of Midwifery, College of Medicine and Health Science, Woldia University, Woldia, Ethiopia; ^6^ Department of Psychiatry, College of Medicine and Health Science, Wollo University, Dessie, Ethiopia

**Keywords:** gestational trophoblastic disease, pregnant women, east africa, prevalence, associated factor

## Abstract

**Background:**

The term of gestational trophoblastic disease (GTD) is used to describe a group of tumors that are characterized by abnormal trophoblastic proliferation. Histologically, GTD includes the pre-malignant partial hydatidiform mole, complete hydatidiform mole, and malignant invasive moles that are choriocarcinoma, placental site trophoblastic tumors, and epithelioid trophoblastic tumors.

**Method and material:**

The protocol for this review was registered on PROSPERO, accessible at CRD42024560408. We used the “PRISMA 2020 Statement: An Updated Guideline for Reporting Systematic Reviews and Meta–analysis. All original and published cross-sectional, cohort, and case-control types of studies were reported during the study period. Studies conducted in both community and institutional settings were considered. Two reviewers independently assessed the risk of bias in the included studies using tools developed by the Joanna Briggs Institute, which comprise eight criteria.

**Result:**

Data was systematically searched by Google Scholar (*n* = 214), HINARI (*n* = 46), Scopus (*n* = 40), and PubMed (*n* = 146) on 1 May 2024. A total of 13 studies from five countries in East Africa have been included. The random effects model showed that the prevalence of GTD among pregnant women was 22% (95% CI: 10%–33%). Women who are aged between 30 and 39 years (AOR = 0.11, 95% CI: 0.05–0.16), women who have a previous history of GTD (AOR = 0.24, 95% CI: 0.00–0.47), women who have a previous complication of the reproductive organ system (AOR = 0.23, 95% CI: 0.10–0.35), and women who have more than two histories of pregnancy (AOR = 0.9, 95% CI: 0.06–0.11) were significantly associated with the outcome variable.

**Conclusion:**

This finding revealed that the pooled prevalence of GTD among pregnant women was high. Women who are aged between 30 and 39 years, have a previous history of GTD, and women who have had previous complications during pregnancy, and women who have more than two histories of pregnancy were significantly associated with the outcome variable.

## Introduction

Gestational trophoblastic disease (GTD) is defined as a spectrum of interconnected conditions but histologically distinct disease entities originating from the placenta (1). It commonly occurs during pregnancy and changes the process and outcome of pregnancy by developing abnormal fertilization and placenta (1).

GTD is used to describe a group of tumors that are characterized by abnormal trophoblastic proliferation. Trophoblastic produces human chorionic gonadotropin, which is why it is important to quantify this peptide for the diagnosis, treatment, and follow-up of this disease (2).

Some evidence shows that GTD most commonly develops after a molar pregnancy; however, it may follow any gestation, including term pregnancy. Histologically, GTD includes the pre-malignant partial hydatidiform mole, complete hydatidiform mole, and malignant invasive moles that are choriocarcinoma, placental site trophoblastic tumors (PSTT), epithelioid trophoblastic tumors (ETT), gestational trophoblastic neoplasm, and invasive mole. choriocarcinoma, PSTT, and ETT are malignant invasive moles that can arise after any type of pregnancy, and all are known as gestational tropgoblastic neoplasia. Therefore, by virtue of their origin, they are able to produce significant amounts of human chorionic gonadotropin (HCG), which is a reliable tumor marker for diagnosis and monitoring of response (3, 4).

Gestational trophoblastic neoplasia is a complication of pregnancy; it may also follow other pregnancy problems like miscarriages, ectopic pregnancy, or term pregnancy. Therefore, by virtue of their origin, they are able to produce a significant amount of HCG, which is a reliable tumor marker for diagnosis and monitoring of response (5–8).

## Method and material

### Registration and protocol

This review was registered by using the protocol on PROSPERO at CRD42024560408. We applied the PRISMA 2020 Statement for Reporting Systematic Reviews and Meta–analysis as a framework (9).

### Eligibility criteria

All original and published cross-sectional and cohort types of study design reporting the magnitude and its determinant factors of GTD in East African countries, systematic review and meta-analysis influencing them were deemed eligible for the systematic review and meta-analysis. Studies conducted in both community and institutional settings were considered. The selection of studies was based on several parameters, including outcome variables, study population, year of the study, regional context, sample size, and response rate. Those studies that do not meet the eligibility criteria are excluded during the search for proper studies. In this research, we included studies that were published from 2015 to 2024.

### Information sources and search strategy

We used Scopus, HINARI, PubMed, and Google Scholar on 1 May 2024 to searching for significant articles for this study, and used searching terms such as “Gestational” AND “trophoblastic” OR “Molar” OR “Hydatidiform” OR “Mole” OR “partial” OR “Complete” “AND “Disease” OR “problem” OR “Infection” OR “Syndrome” AND “Magnitude” AND “Determinant” “AND” Prevalence “OR” associated “AND “Factors” AND “pregnancy” OR “mother” AND “East” AND “Africa.” Manual searching was performed on PubMed, HINARI, Scopus, and Google Scholar by using the “Publish or Perish” database searching tool, version 8, to determine the published and unpublished articles (10).

### Selection process

The remaining studies had been separately screened by NA and MA after duplicate studies were removed with EndNote 20. These researchers performed the selection process accurately. Articles were initially improved on the basis of their abstract and title; full-text modifications followed, first alternately and then collectively until a consensus had been reached. Third reviewer, GB, was consulted for an agreement in circumstances wherever there was disagreement.

### Data collection process and data items

For data extraction, a Microsoft Excel 2019 spreadsheet was used. This spreadsheet was utilized to extract the following outcome variables: population (study units), year of study, context, sample size, response rate, and proportions. Following the extraction of the data and comparing their findings, the two independent reviewers, NA and MA, came to an agreement. While there was unwilling to reach agreement to a consensus, GB, a third reviewer, was requested to provide assistance. Associated factors of GTD in pregnant women in East Africa were the main outcomes of this comprehensive review and meta-analysis. Other factors influencing the primary result variables were incorporated in the additional outcomes.

### Study risk of bias assessment

Two reviewers, NA and MA, independently assessed the risk of bias in the included studies using tools developed by the Joanna Briggs Institute, which comprise eight criteria. The assessment focused on several aspects: inclusion in the sample, descriptions of study subjects and settings, validity and reliability of measurements, confounding factors and strategies to address them, and appropriateness of the outcome measures. Scores of 7 or higher were classified as low risk, 5–6 as medium risk, and 4 or lower as high risk. Studies identified as low- and medium-risk were then included in the review. Depicts that the average risk of bias across the studies was 5.76, representing 72% (**Table 1**).

**Table 1:** Summary of the risk of bias assessment of the included studies (n = 13), 2024.

Study ID	Clear sampling criteria	Participants and setting	Measurement of exposure	Objective and/or question	Identification of confounding factors	Strategies to deal with confounding factors	Measurement of outcome	Appropriate statistical analysis	Score	Risk
Yilma.M(2020) (1)	**+**	**+**	**?**	**+**	**?**	**+**	**+**	**+**	6/8	Medium
Barkadle.A(2022) (25)	**+**	**+**	**+**	**+**	**?**	**+**	**+**	**+**	7/8	Low
Kahsay.H(2019) (26)	**+**	**+**	**+**	**+**	**?**	**?**	**+**	**?**	5/8	Medium
Ahmed.Y(2019) (27)	**+**	**+**	**+**	**+**	**?**	**?**	**+**	**+**	6/8	Low
Amina. R(2024) (28)	**+**	**+**	**+**	**+**	**?**	**-**	**+**	**+**	6/8	Medium
Chesrem.E (29)	**+**	**+**		**+**	**?**	**?**	**+**	**+**	5/8	Medium
Gitau.S (30)	**+**	**+**	**+**	**+**	**-**	?	**+**	**+**	6/8	Medium
Wekesa.A (31)	**+**	**+**	**+**	**+**			**+**	**?**	5/8	Medium
Ariggah (32)	**+**	**+**	**+**	**+**			**+**	**+**	6/8	Medium
B.Kitange (33)	**+**	**+**	**+**	**+**			**+**	**+**	6/8	Medium
MwajumaB (3)	**+**	**+**	**+**	**+**			**+**	**+**	6/8	Medium
Mulisya.O (34)	**+**	**+**	**+**	**+**			**+**	**?**	5/8	Medium
Al Riyami,N (35)	**?**	**?**	**+**	**+**			**+**	**+**	6/8	Medium
**Average**									**5.76/8**	**Medium**

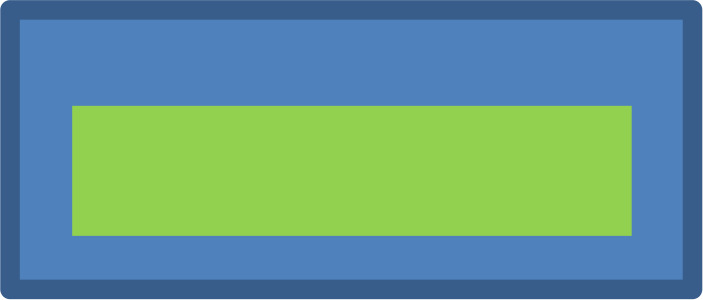
, **Low risk**  
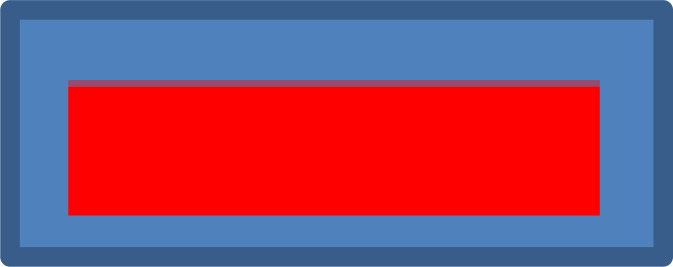
, **High risk**  
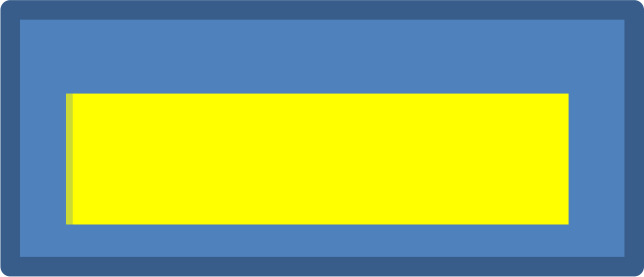
, **Unclear**.

### Effect measures and synthesis methods

The outcome variables were conceptually categorized for the qualitative synthesis using thematic approaches. Using a Microsoft Excel 2019 spreadsheet, preliminary effect measurements for the quantitative synthesis have been determined based on the qualitative synthesis. The effect estimates (proportions and odds ratios, or ORs) of the determinants of GTD in pregnant women were calculated by using STATA 17. Regardless of their significance levels, we included the associated variables, which were classified. These effect estimates were then compared among studies that targeted the outcome variables using subgroup statistical analysis. A 95% confidence interval (CI) and a p-value of less than 0.05 were taken to be the cutoff point for overall statistical significance.

### Reporting bias and certainty assessment

The *I* ^2^ statistic was used to evaluate study heterogeneity. By comparing the effect estimates (proportions and ORs) of the determinants of gestational trophoblastic illness between countries and based on research design, percentages of weights and subgroup analysis were used to gauge each study’s contribution to the overall meta-analysis. The outlier studies were also identified through a sensitivity analysis. Additionally, any inter-study bias was investigated using Doi plots.

## Result

### Study selection

**Figure 1:**
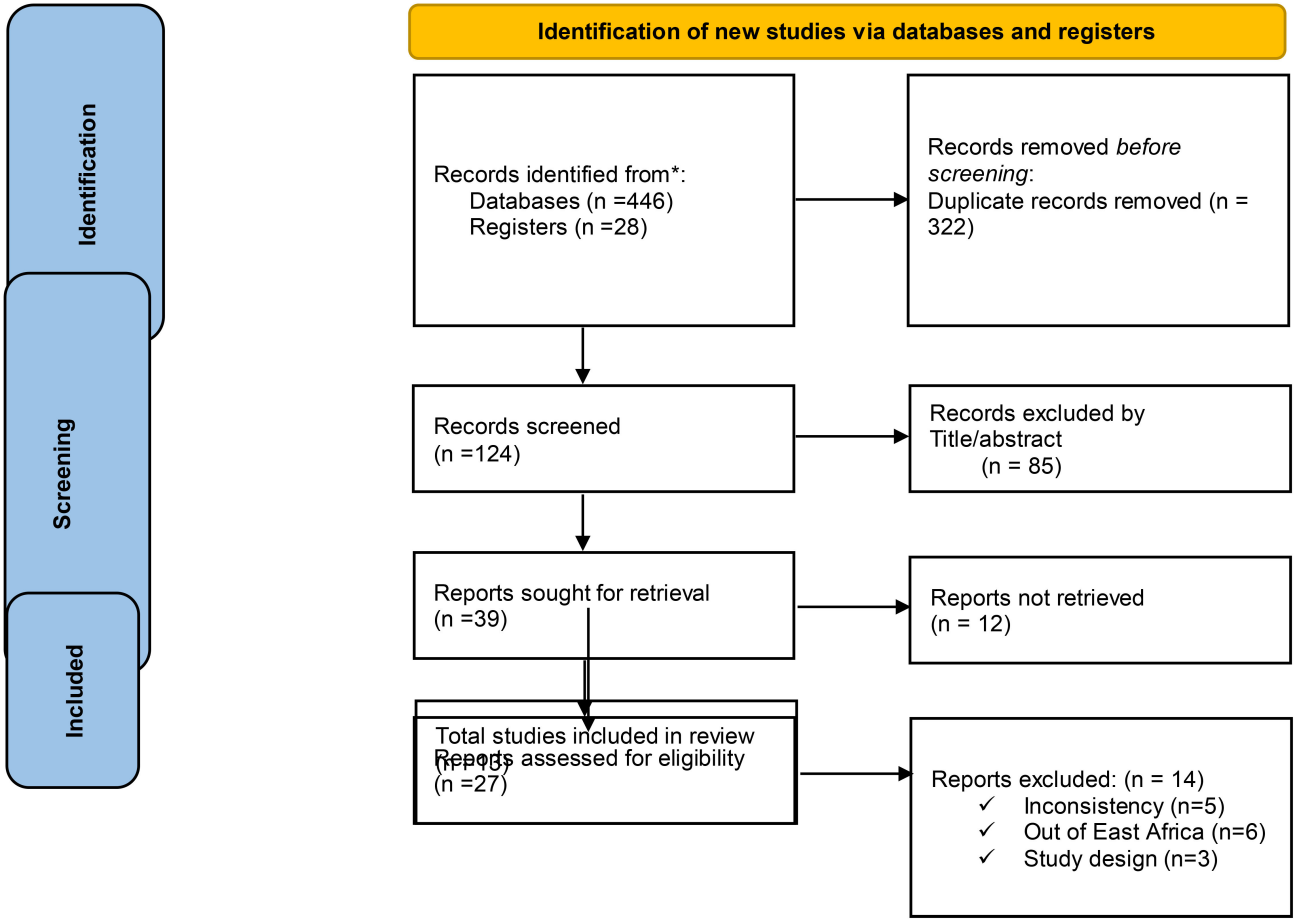
PRISMA flow diagram of study selection process.

We systematically searched Google Scholar (*n* = 214), HINARI (*n* = 46), Scopus (*n* = 40), and PubMed (*n* = 146) on 1 May 2024. Additionally, 28 records were found through other sources, resulting in a total of 474 resources being searched. Following the removal of duplicates, 322 articles remained. Then, after excluding 124 resources through relevance, 85 articles were screened for title and abstract evaluation, which resulted in the exclusion of 39 resources by resource sought for retrieval (27 researches not fully filling the eligibility criteria and 12 researches by reports which were not retrieved) by full text review of each article. After the full text review of each article, 13 resources were identified for inclusion, all of that were considered suitable for the quantitative meta-analysis (**Figure 1**).

### Qualitative synthesis

#### Characteristics of individual studies and participants pertain to GTD among pregnant women

A total of 13 studies from five countries in East Africa have been included [from Ethiopia (*n* = 4), Kenya (*n* = 5), Tanzania (*n* = 2), Uganda (*n* = 1), and Somalia (*n* = 1)]. Data were synthesized regarding the magnitude and its associated factors of GTD among pregnant women. Among the 13 studies, the majority (*n* = 9) were conducted by a cross-sectional study design, while the remainder (*n* = 4) were carried out by cohort types of study design (**Table 2**).

**Table 2:** Characteristics of individual Studies and participants pertain for gestational trophoblastic disease among pregnant women.

Study ID	Design	Publication year	Countries	Sample size	Magnitude	*P*-value
Yilma.M (1)	Cross-sectional	2020	Ethiopia	16,957	194	0.0114
Barkadle.A (25)	Cross-sectional	2022	Ethiopia	17,201	181	0.0105
Kahsay.H (26)	Cross-sectional	2019	Ethiopia	4802	457	0.290
Ahmed.Y (27)	Cross-sectional	2019	Ethiopia	11,453	83	0.01
Amina. R (28)	Cross-sectional	2024	Kenya	155	98	0.632258
Chesrem.E (32)	Cross-sectional	2019	Kenya	250	48	0.192
Gitau.S (30)	Cohort	2017	Kenya	158	103	0.6515
Wekesa.A (31)	Cohort	2021	Kenya	110	37	0.3365
Riggah (29)	Cohort	2020	Kenya	85	11	0.129
B.Kitange (33)	Cross-sectional	2015	Tanzania	180	23	0.128
Mwajuma.B (3)	Cross-sectional	2023	Tanzania	200	42	0.21
Mulisya.O (34)	Cross-sectional	2018	Uganda	181	11	0.061
Al Riyami,N (36)	Cohort	2019	Somalia	64	19	0.03

#### Prevalence of GTD among pregnant women

The estimated prevalence and ranges of GTD among pregnant women was reported by using a random effects model in East African countries. Double arcsine transformation was used to normalize the distribution of the effect size. This review revealed that there is a high prevalence of GTD among pregnant women in studies performed in Kenya, 65% (5% CI: 58%–73%), and this prevalence has declined in studies performed in Ethiopia, 0.1% (95% CI: 0.01–0.01).

**Figure 2:**
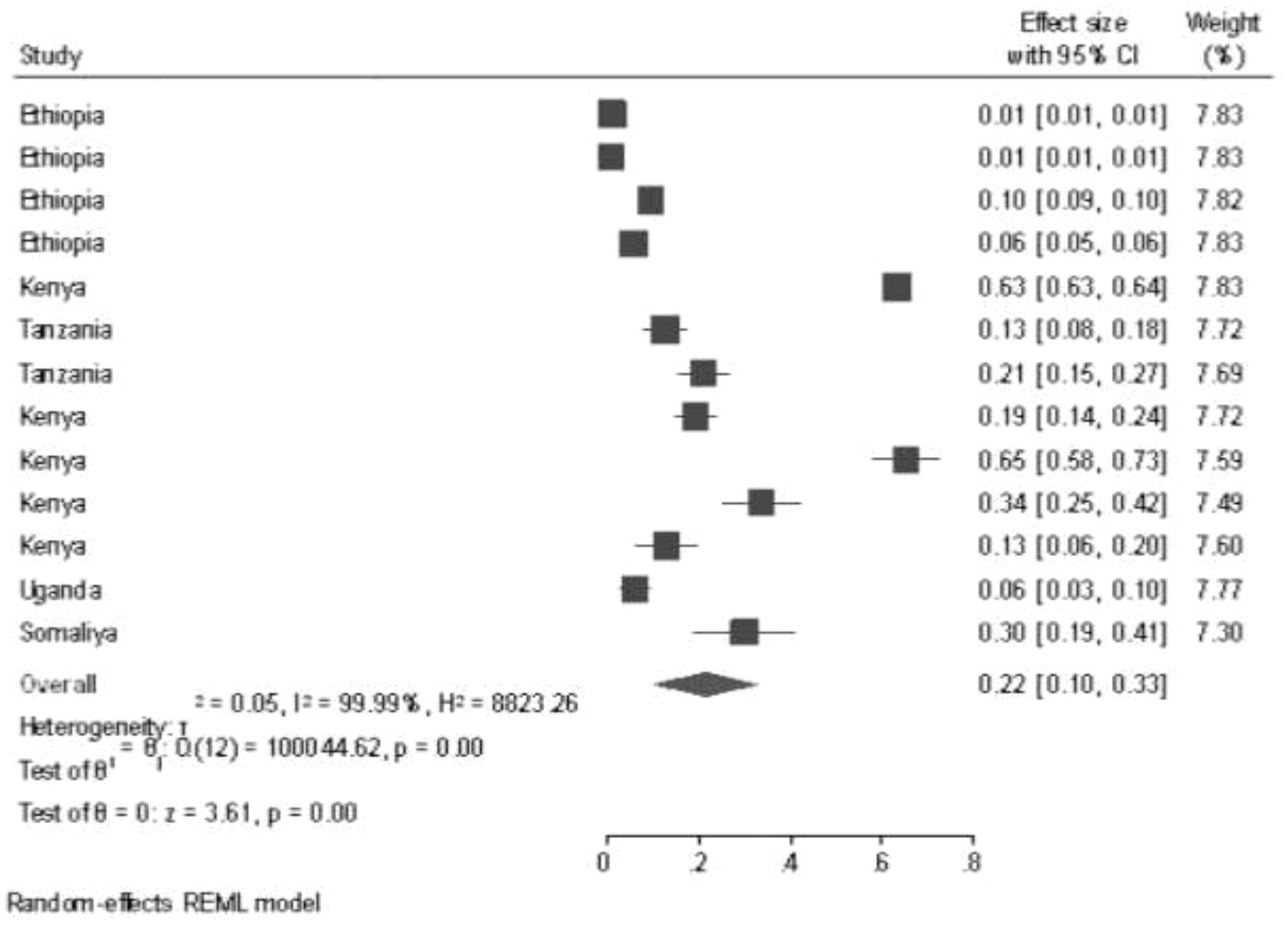
Pooled prevalence of gestational trophoblastic disease among pregnant women.

The random effects model showed that the pooled prevalence of GTD among pregnant women was 22% (95% CI: 10%–33%), and there was significant heterogeneity among the studies (*I* ^2^ = 99.99%, *p* < 0.001) (**Figure 2**).

### Subgroup analysis and investigation of heterogeneity

**Figure 3:**
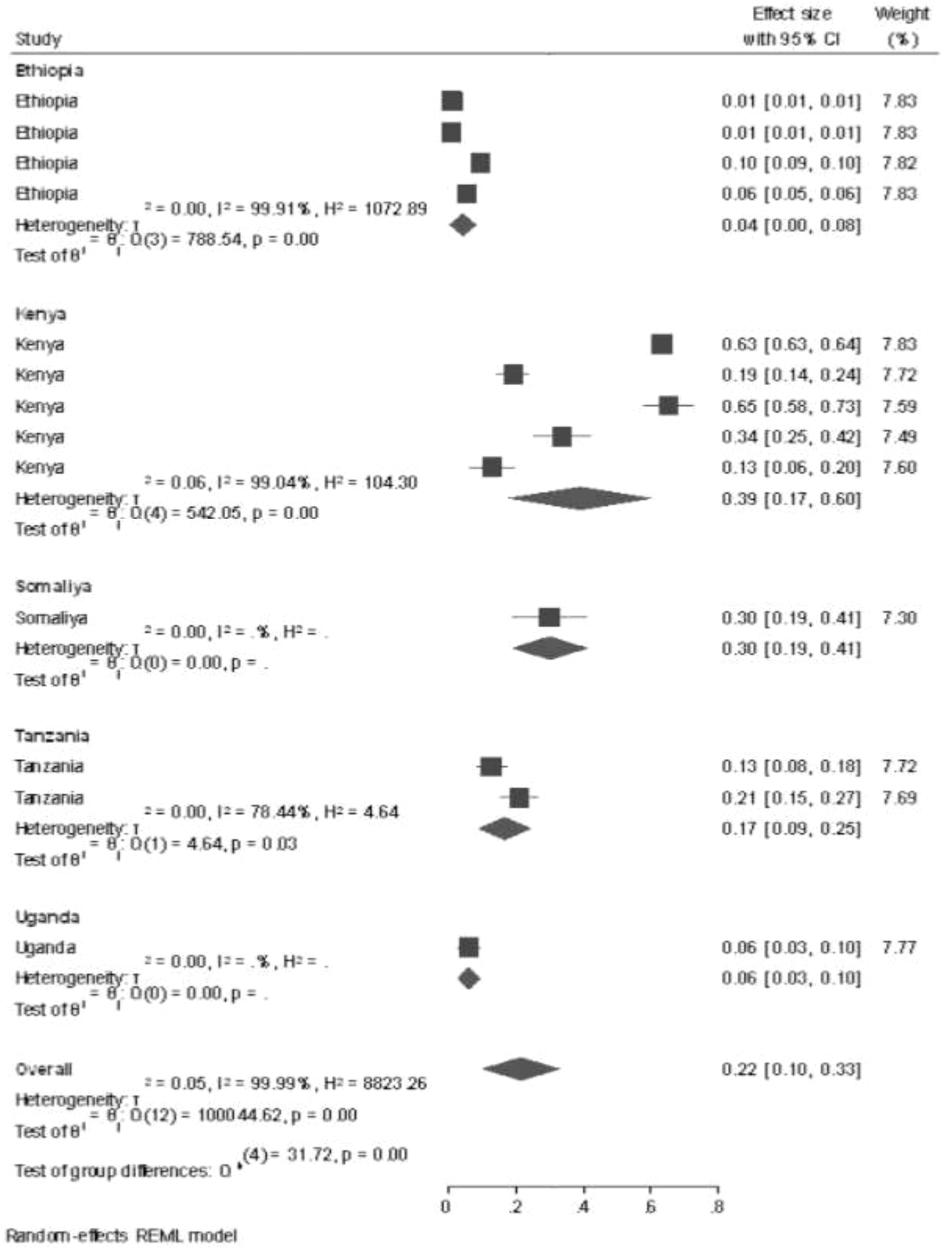
Subgroup analysis and investigation of heterogeneity.

Subgroup analysis by country for all studies performed in East Africa was conducted by using a systematic review and meta-analysis method, and the prevalence of GTD among pregnant women was highest in the study conducted in Kenya (39%, 95% CI: 17%–60%), followed by Somalia (30%, 95% CI: 19%–41%), and the lowest prevalence of GTD among pregnant women was shown in the study conducted in Ethiopia (0.4%, 95% CI: 0.00%–0.8%) and 2015 (41%, 95% CI: 38%–45%). In the sub-country analysis, potential heterogeneity was detected between each study in the prevalence estimates of GTD among pregnant women (*I* ^2^ = 99.99%; all *p* < 0.001). This potential heterogeneity took place using different sample sizes, sampling techniques, and different study populations (**Figure 3**).

### Publication bias of the study

**Figure 4:**
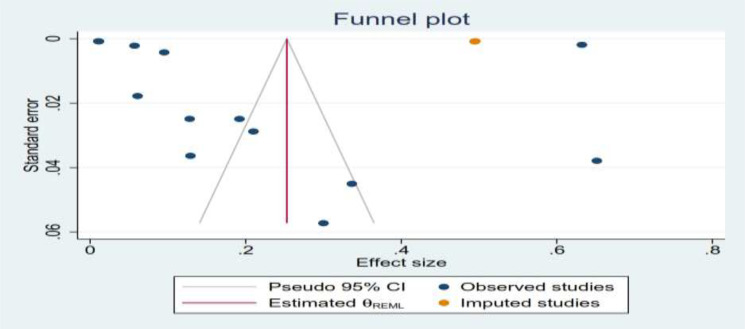
Publication bias about gestational trophoblastic disease among pregnant women.

Publication bias was assessed via visual inspection of funnel plots. Funnel plot analysis revealed that there were asymmetrical studies, which revealed publication bias between each study, but the publication bias analysis showed the test provides that there is evidence for the presence of small-study effects of the funnel plot; this effect takes place due to using of different studies done by different sample sizes, study populations, and different sampling techniques (**Figure 4**).

### Sensitivity analysis

After assessing the variability effects between each study by considering the subgroup analysis and publication bias (*I* ^2^ = 99.99%; all *p* < 0.001), there was detection of heterogeneity between each studies; therefore, a sensitivity analysis was done by using a trim and fill analysis to detect the studies that affect the variability effect between each variable. The nonparametric trim-and-fill analysis of publication bias showed that the heterogeneity effects were detected by omitting one study, which was done in Kenya, from the total findings (**Table 3**).

**Table 3:** Sensitivity analysis of gestational trophoblastic disease among pregnant women.

Studies	Effect size	[95% confidence interval]
Observed	0.215	[0.098–0.332]
Observed + imputed	0.215	[0.098–0.332]

### Associated factors of GTD among pregnant women

In this study, women who are aged between 30 and 39 years, the occurrence of complications during pregnancy, women who have more than two pregnancies, and women who have a previous history of GTD were significantly associated with GTDs during the pregnancy period.

In this study, women who were aged between 30 and 39 years were 11% less likely to develop GTD during pregnancy than those aged less than 30 years (AOR = 0.11, 95% CI: 0.05–0.16). Similarly, women who have a previous history of GTD were 24% less likely to develop GTD than those with no history of GTD during the pregnancy period (AOR = 0.24, 95% CI: 0.00–0.47).

Women who have previous complications of the reproductive organ system were 77% less likely to develop GTD during pregnancy (AOR= 0.23, 95% CI: 0.10–0.35). Women who have more than two histories of pregnancy were 91% less likely to develop GTD than those with a history of less than two pregnancies (AOR = 0.9, 95% CI: 0.06–0.11) (**Table 4**).

**Table 4:** Associated factors of gestational trophoblastic disease among pregnant women. .

Variables		Gestational trophoblastic diseases		AOR (95% CI)
		Yes	No	
Age of the women	20–29 years 2086 30–39 years 10210 40–49 years 4520	616 (3.664%)	16200(96.336%)	10.11 (95% CI: 0.05–0.16)
History of complication	Yes 12,756 No 34,238	850 (1.809%)	46144(98.191%)	0.24 (95% CI: 0.00–0.47) 1
Previous history of GTD	Yes 6520 No 27998	460(1.3264%)	34058 (98.673%)	0.23 (95% CI: 0.10–0.35) 1
Gravidity	1–2 pregnancies 8618 > 2 pregnancies 8083	585 (3.502%)	16116 (96.498%)	1 (0.9 (95% CI: 0.06–0.11).

## Discussion

In this study, the finding revealed that the pooled prevalence of GTD among pregnant women was 22% (95% CI: 10%–33%). This finding was similar to the study conducted in Egypt (11). But this study was higher than the study conducted in Tunisia (12), Egypt, Sindh (13), Enugu, Zaria (15), Kuanz-natal (16), Parma (17), Madhya (18), Odisha (19), and Mysore (20). But this finding was lower than the study conducted in Rajasthan (21) and Hyderabad (22). These differences might be due to study setting, healthcare access, diagnostic practices, and socio-demographic factors of each study.

In this finding age of the women between 30 and 39 years, complication history during the pregnancy period, previous history of GTD, and women who had more than two pregnancies were significantly associated with GTD during their pregnancy period.

In this finding, the ages of women between 30 and 39 years are highly risk factors for GTD; this finding was in line with studies conducted in Enugu, Nigeria (14), Tunisia (12), and Lower Egypt (11).

This study showed that women who have a previous history of GTD were significantly associated with GTD during her pregnancy period; this finding was in line with a study conducted in Qazvin (Iran) (23).

This finding showed that a history of complications was significantly associated with the GTD among women during the pregnant period; this finding was in line with a study conducted in Tehran, Iran (24).

This finding showed that gravity greater than 2 was significantly associated with the occurrence of GTD among pregnant women. This finding was congruent with a study conducted in Qazvin (Iran) (23); it is different from a study conducted in Indore, Madhya (18). This might be due to the socio-demographic status of the participants, types of study methodology, and study area of the participants, and this difference could be that the current study included pooled data from different East African countries.

Subgroup analysis by country for all studies performed in East Africa was conducted by using a systematic review and meta-analysis method, and the prevalence of GTD among pregnant women was highest in the study conducted in Kenya (39%, 95% CI: 17%–60%), followed by Somalia (30%, 95% CI: 19%–41%), and the lowest prevalence of GTD among pregnant women was shown in the study conducted in Ethiopia (0.4%, 95% CI: 0.00%–0.8%) and 2015 (41%, 95% CI: 38%–45%). The possible explanation of the difference might be due to the number of studies, types of study design, and sample size determinations; these are some factors affecting the sub-group results.

## Limitation of the study

This study used only studies performed in East African countries. This study lacks comparator reviews done by systematic review and meta-analysis; we discussed the results of our systematic and meta-analysis against original studies conducted in various other countries out of the context area.

## Conclusion

In this study, the finding revealed that the pooled prevalence of GTD among pregnant women was high compared with other African regions.

Women who are aged between 30 and 39 years, women with a previous history of GTD, women who have a previous complication of the reproductive organ system, and women who have more than two histories of pregnancy were significantly associated with the outcome variable. Therefore, all women should have preconception care before pregnancy and proper ANC follow-up during the pregnancy period, and also all concerned bodies should put their own efforts to eliminate the complication of the problem during the pregnancy period.
